# Clinical outcomes in transplant‐eligible patients with relapsed or refractory diffuse large B‐cell lymphoma after second‐line salvage chemotherapy: A retrospective study

**DOI:** 10.1002/cam4.6412

**Published:** 2023-08-28

**Authors:** Yu Yagi, Yusuke Kanemasa, Yuki Sasaki, Mina Sei, Takuma Matsuo, Kento Ishimine, Yudai Hayashi, Mano Mino, An Ohigashi, Yuka Morita, Taichi Tamura, Shohei Nakamura, Toshihiro Okuya, Takuya Shimizuguchi, Naoki Shingai, Takashi Toya, Hiroaki Shimizu, Yuho Najima, Takeshi Kobayashi, Kyoko Haraguchi, Noriko Doki, Yoshiki Okuyama, Tatsu Shimoyama

**Affiliations:** ^1^ Department of Medical Oncology, Tokyo Metropolitan Cancer and Infectious Diseases Center Komagome Hospital Tokyo Japan; ^2^ Department of Pharmacy, Tokyo Metropolitan Cancer and Infectious Diseases Center Komagome Hospital Tokyo Japan; ^3^ Department of Radiation Oncology, Tokyo Metropolitan Cancer and Infectious Diseases Center Komagome Hospital Tokyo Japan; ^4^ Hematology Division, Tokyo Metropolitan Cancer and Infectious Diseases Center Komagome Hospital Tokyo Japan; ^5^ Division of Transfusion and Cell Therapy, Tokyo Metropolitan Cancer and Infectious Diseases Center Komagome Hospital Tokyo Japan

**Keywords:** autologous stem cell transplantation, chimeric antigen receptor T‐cell therapy, relapsed or refractory diffuse large B‐cell lymphoma, salvage chemotherapy

## Abstract

**Objective:**

The prognosis of patients with relapsed or refractory (R/R) diffuse large B‐cell lymphoma (DLBCL) is poor. Although patients who fail first‐line salvage chemotherapy are candidates for second‐line salvage chemotherapy, the optimal treatment strategy for these patients has not yet been established.

**Methods:**

The present, single‐center, retrospective study included transplant‐eligible patients with R/R DLBCL who received second‐line salvage chemotherapy with curative intent.

**Results:**

Seventy‐six patients with R/R DLBCL received second‐line salvage chemotherapy. Eighteen (23.7%) patients were responders to the first‐line salvage chemotherapy. The overall response rate was 39.5%, and overall survival (OS) was significantly longer in patients who responded to second‐line salvage chemotherapy than those who did not. Forty‐one patients who proceeded to potentially curative treatment (autologous hematopoietic stem cell transplantation [ASCT], chimeric antigen receptor [CAR] T‐cell therapy, or allogeneic hematopoietic stem cell transplantation) had a better prognosis than those who did not. Among the 46 patients who failed to respond to the second‐line salvage regimen, only 18 (39.1%) could proceed to the curative treatments. However, among the 30 patients who responded to the second‐line salvage regimen, 23 (76.7%) received one of the potentially curative treatments. Among 34 patients who received CAR T‐cell therapy, OS was significantly longer in those who responded to salvage chemotherapy immediately prior to CAR T‐cell therapy than in those who did not respond. In contrast, the number of prior lines of chemotherapy was not identified as a statistically significant prognostic factor of survival. No significant difference was detected in OS between patients receiving ASCT and those receiving CAR T‐cell therapy after the response to second‐line salvage chemotherapy.

**Discussion:**

In this study, we demonstrated that chemosensitivity remained a crucial factor in predicting survival outcomes following CAR T‐cell therapy irrespective of the administration timing, and that both ASCT and CAR T‐cell therapy were acceptable after the response to second‐line salvage chemotherapy.

## 1 INTRODUCTION

Diffuse large B‐cell lymphoma (DLBCL) is the most common subgroup of lymphoma and accounts for 30%–40% of all adult lymphomas.^1^, ^2^ The addition of rituximab to the CHOP (cyclophosphamide, doxorubicin, vincristine, and prednisone) regimen (R‐CHOP) starting in the late 1990s has improved the survival outcomes of patients with DLBCL.^3^, ^4^, ^5^, ^6^ Currently, R‐CHOP or similar regimens are considered standard treatment for DLBCL. While DLBCL is potentially curable, approximately one third of patients will ultimately develop relapsed or refractory (R/R) disease after first‐line chemotherapy.^7^, ^8^

The standard treatment option in chemotherapy‐sensitive R/R DLBCL is high‐dose chemotherapy followed by autologous stem cell transplantation (ASCT).^9^, ^10^ However, owing to advanced age or coexisting medical conditions, only half the patients on this treatment pathway are candidates for transplantation. Moreover, only about half the patients respond to the initial salvage therapy and qualify for ASCT, which has an overall cure rate of 25%–35%.^9^, ^11^ Nonetheless, intensive chemotherapy followed by ASCT remains an option even when patients are not sufficiently responsive to the first‐line salvage chemotherapy, and further salvage chemotherapies are needed for disease reduction.^12^

The therapeutic efficacy of chimeric antigen receptor (CAR) T‐cell therapy has drastically changed the treatment strategy of R/R DLBCL.^13^ In Japan, CAR T‐cell therapy has been approved for use in patients with R/R DLBCL who have failed at least two lines of systemic therapy. In the standard care setting, a wide variety of salvage chemotherapies are administered before CAR T‐cell therapy.^14^ However, a substantial proportion of patients considered eligible for CAR T‐cell therapy fail to undergo CAR T‐cell infusion because of rapid DLBCL progression.^14^, ^15^ Therefore, effective salvage chemotherapies are required to control R/R DLBCL growth during the time required to manufacture the CAR T‐cells and perform their infusion.

The rate of response to first‐line salvage chemotherapy regimens ranged from 45% to 65%, with no clear difference between regimens.^9^, ^11^, ^16^ The response rate to second‐line salvage chemotherapy was even lower at 13.7%–52% in patients with an insufficient response to first‐line salvage therapy.^17^, ^18^, ^19^, ^20^, ^21^, ^22^, ^23^ The response to salvage chemotherapy is critical for administering the subsequent, potentially curative therapies, namely ASCT and CAR T‐cell therapy. However, the optimal management strategy for patients who are unresponsive to first‐line salvage chemotherapy is yet to be established.

The present study evaluated the efficacy of second‐line salvage chemotherapy and subsequent treatments among transplant‐eligible patients with R/R DLBCL.

## 2 METHODS

### 2.1 Patients

The present study was a retrospective review of the medical records of ASCT‐eligible patients with R/R DLBCL who received second‐line salvage chemotherapy with curative intent at Tokyo Metropolitan Cancer and Infectious Diseases Center, Komagome Hospital between June 2006 and January 2023. Patients with central nervous system (CNS) involvement were excluded.

A pathology review based on the World Health Organization classification was performed by hematopathologists at our hospital.^1^, ^2^ Cells of origin were identified with the Hans algorithm, which uses the immunohistochemical expression of three markers (CD10, BCL6, and MUM1).^24^ Performance status (PS) was evaluated using the Eastern Cooperative Oncology Group (ECOG) criteria and clinical stage was assessed using the Ann Arbor classification. The International Prognostic Index (IPI) score was calculated based on age, PS, clinical stage, serum lactate dehydrogenase (LDH), and extranodal involvement at diagnosis or at second‐line salvage chemotherapy.^25^

### 2.2 Salvage chemotherapy

The salvage regimens used in this study were based on etoposide, ifosfamide, cis‐ or carbo‐platin, bendamustine, cytarabine, or gemcitabine. The regimens listed in Table S1 were classified as salvage chemotherapy.^26^, ^27^, ^28^, ^29^, ^30^, ^31^, ^32^, ^33^, ^34^, ^35^, ^36^, ^37^, ^38^, ^39^, ^40^ Rituximab was administered concurrently in most of the patients. Response to the therapy was assessed by computed tomography (CT) and/or positron‐emission tomography (PET)‐CT in accordance with the International Working Group response criteria.^41^

### 2.3 Statistical analysis

Time‐to‐event endpoints were evaluated using the Kaplan–Meier method, with differences between groups compared using the log‐rank test. Overall survival (OS) was defined as the period from the date of the first cycle of second‐line salvage chemotherapy to the date of last follow‐up or death from any cause. Progression‐free survival (PFS) was defined as the period from the date of the first cycle of second‐line salvage chemotherapy to the date of the last follow‐up, documented progression, relapse, or death from any cause. Multivariate analysis was performed for PFS and OS using the Cox proportional hazards model. Independent variables with at least marginal association with PFS/OS in univariable analysis (*p* < 0.05) were included in the multivariable analysis. Categorical variables were evaluated using Fisher's exact test. All statistical analyses were performed with R software (version 4.2.2) or EZR software (version 1.41).^42^ Two‐tailed *p* < 0.05 was considered to indicate statistical significance.

## 3 RESULTS

### 3.1 Patient characteristics

**FIGURE 1:**
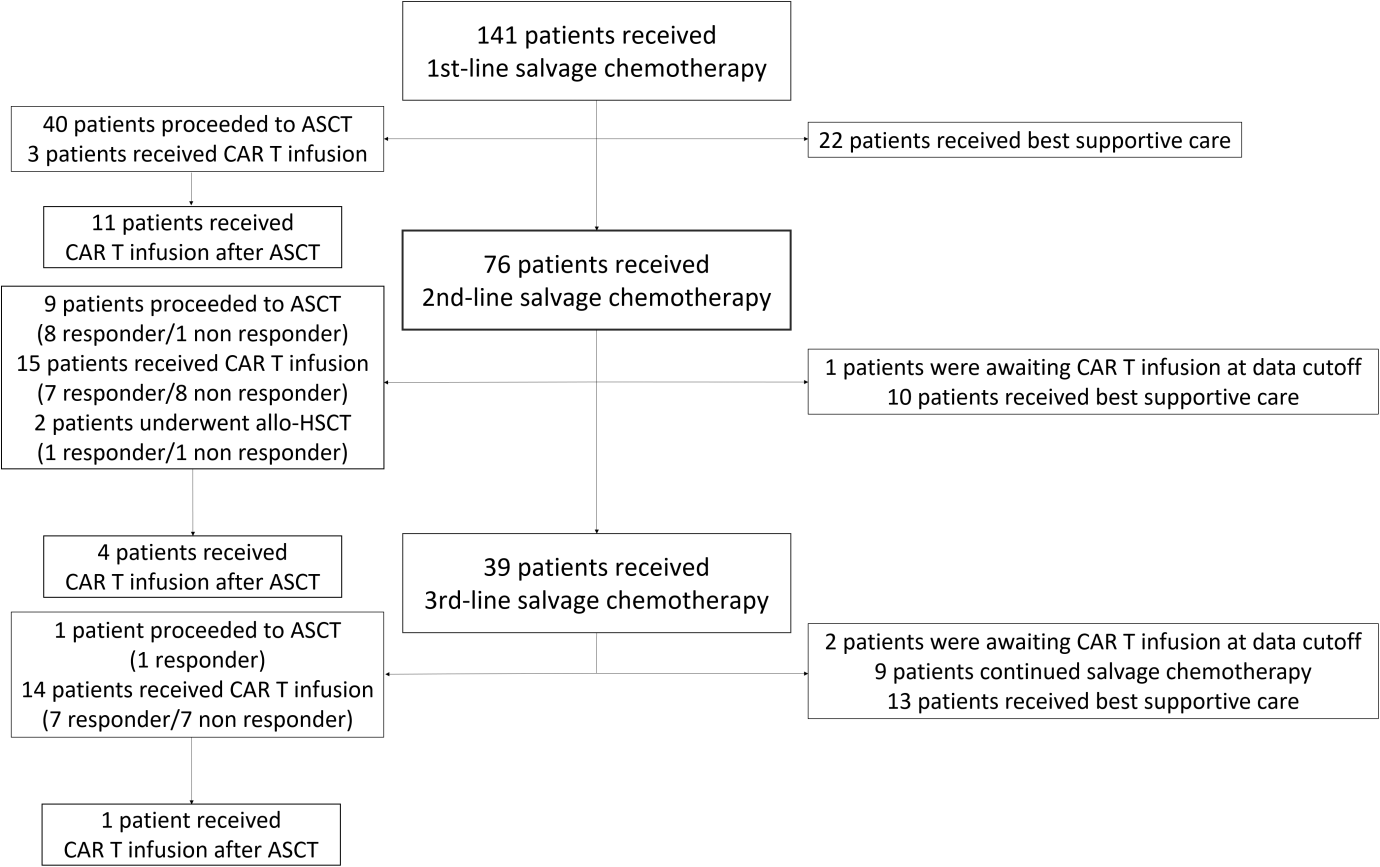
Flow chart of the patients in the study cohort.

Seventy‐six of 141 patients with R/R DLBCL who received first‐line salvage chemotherapy were treated by second‐line salvage chemotherapy with curative intent (Figure 1). Table 1 shows the patient characteristics. All 76 patients had received R‐CHOP‐based therapy as their first‐line chemotherapy, with initial overall response rate (ORR) of 71.1% (28.9% CR and 42.1% PR). Sixty‐two (81.6%) patients had refractory disease or a relapse less than 12 months after diagnosis. All the 76 patients received a median of two cycles of the first‐line salvage chemotherapy, mostly Ara‐C based regimens (range: 1–6). While 58 (76.3%) patients were not responders to first‐line salvage chemotherapy, 18 (23.7%) patients responded to the first‐line salvage chemotherapy; of the 18 patients, four had a response which was considered inadequate for proceeding to ASCT and still had bulky disease. Fourteen patients did not receive ASCT because of complications, an insufficient stem cell harvest or other reasons.

**TABLE 1:** Characteristics of patients with relapsed/refractory non‐Hodgkin B‐cell lymphoma receiving second‐line salvage chemotherapy.

Characteristic	No. (%)
No. of patients	76
Median age at diagnosis (range in years)	55 (17–73)
Sex (male)	47 (61.8)
ECOG PS at diagnosis	
0–1	65 (85.5)
≥2	11 (14.5)
Diagnosis	
DLBCL, NOS	56 (73.7)
HGBCL	3 (3.9)
PMBCL	5 (6.6)
IVLBCL	1 (1.3)
TFL	11 (14.5)
Double‐ or triple‐hit rearrangement: MYC plus BCL2, BCL6, or both; No./total No. (%)	3/30 (9.1)
Cell of origin	
GCB	36 (52.2)
Non‐GCB	33 (47.8)
Missing data	7
Bulky disease (≥10 cm) at diagnosis	26/75 (34.7)
Bone marrow involvement at diagnosis	16 (21.1)
Disease stage at diagnosis	
I or II	20 (26.3)
III or IV	56 (73.7)
Extranodal involvement at diagnosis	
0–1	46 (60.5)
≥2	30 (39.5)
International Prognostic Index score at diagnosis	
0–2	32 (50)
3–5	32 (50)
Missing or incompletely assessed	12
First‐line chemotherapy	
R‐CHOP	60 (79)
Dose‐adjusted‐EPOCH‐R	11 (14.5)
HyperCVAD/MA	1 (1.3)
R‐CHOP‐like	4 (5.1)
Relapsed or refractory	
Relapsed, >12 month	14 (18.4)
Refractory, <12 months	62 (81.6)
First‐line salvage chemotherapy	
AraC‐based regimen	
ESHAP	26 (34.2)
CHASE	22 (28.9)
DHAP	1 (1.3)
ACES	6 (7.9)
HD‐MA	1 (1.3)
HyperCVAD/MA	1 (1.3)
Ifosfamide‐based regimen	
ICE	3 (3.9)
DeVIC	1 (1.3)
Gemcitabine‐based regimen	
GDP	7 (9.2)
GCD	3 (3.9)
Other	
Pola‐BR	3 (3.9)
CHOP‐like	1 (1.3)
HD‐MTX	1 (1.3)
Median cycles (range)	2 (1–6)
Rituximab in first‐line salvage chemotherapy	67 (88.2)
Response to first‐line salvage chemotherapy	
Complete response or partial response	18 (23.7)
Stable disease or progressive disease	58 (76.3)
Radiotherapy after first‐line salvage chemotherapy	8 (10.5)

Abbreviations: ACES, high‐dose cytarabine, etoposide, carboplatin, and solumedrol; CHASE, high‐dose cytarabine, cyclophosphamide, etoposide, and dexamethasone; CHOP, cyclophosphamide, doxorubicin, vincristine, and prednisone; DeVIC, carboplatin, etoposide, ifosfamide, and dexamethasone; DHAP, high‐dose cytarabine, cisplatin, and dexamethasone; DLBCL, diffuse large B‐cell lymphoma; ECOG PS, Eastern Cooperative Oncology Group performance status; EPOCH, etoposide, doxorubicin, vincristine, cyclophosphamide, and prednisone; ESHAP, high‐dose cytarabine, cisplatin, etoposide, and methylprednisolone; GCB, germinal center B‐cell type; GCD, gemcitabine, carboplatin, and dexamethasone; GDP, gemcitabine, cisplatin, and dexamethasone; HD‐MA, high‐dose methotrexate/cytarabine; HD‐MTX, high‐dose methotrexate; HGBCL, high‐grade B‐cell lymphoma; Hyper CVAD/MA, cyclophosphamide, doxorubicin, vincristine, dexamethasone, methotrexate and high‐dose cytarabine; ICE, carboplatin, etoposide, and ifosfamide; IVLBCL, intravascular large B‐cell lymphoma; NOS, not otherwise specified; PMBCL, primary mediastinal large B‐cell lymphoma; Pola‐BR, polatuzumab vedotin, bendamustine, and rituximab; R, rituximab; TFL, transformed follicular lymphoma.

### 3.2 Response to second‐line salvage chemotherapy

Various chemotherapy regimens were used as second‐line salvage chemotherapy (Table 2). Patients received a median of two cycles (range: 1–8). Following the second‐line salvage chemotherapy, 30 patients (39.5%) had achieved CR or PR, 17 (22.4%) had SD, and 29 (38.2%) showed disease progression (Table 2). A total of 26 patients received potentially curative treatments after second‐line salvage chemotherapy, nine ASCT (8/9 responders), 15 CAR‐T (7/15 responders), and two allogeneic hematopoietic stem cell transplantation (allo‐HSCT) (1/2 responders) (Figure 1).

**TABLE 2:** Details of second‐line salvage chemotherapy.

Characteristic	No. (%)
No. of patients	76
Second‐line salvage chemotherapy	
AraC‐based regimens	
ESHAP	4 (5.3)
CHASE	13 (17.1)
DHAP	2 (2.6)
IVAC	1 (1.3)
Ifosfamide‐based regimen	
ICE	10 (13.2)
DeVIC	18 (23.7)
IVAM	2 (2.6)
Gemcitabine‐based regimen	
GDP	8 (10.5)
GCD	5 (6.6)
Other	
Dose‐adjusted‐EPOCH	1 (1.3)
EPOCH	2 (2.6)
Pola‐BR	9 (11.8)
Others	1 (1.3)
Median cycles (range)	2 (1–8)
Rituximab in second‐line salvage chemotherapy	71 (93.4)
Response to second‐line salvage chemotherapy	
Complete response or partial response	30 (39.5)
Stable disease or progressive disease	46 (60.5)
Received potentially curative treatments after second‐line salvage chemotherapy	26 (34.2)

Abbreviations: CHASE, high‐dose cytarabine, cyclophosphamide, etoposide, and dexamethasone; DeVIC, carboplatin, etoposide, ifosfamide, and dexamethasone; DHAP, high‐dose cytarabine, cisplatin, and dexamethasone; EPOCH, etoposide, doxorubicin, vincristine, cyclophosphamide, and prednisone; ESHAP, high‐dose cytarabine, cisplatin, etoposide, and methylprednisolone; GCD, gemcitabine, carboplatin, and dexamethasone; GDP, gemcitabine, cisplatin, and dexamethasone; ICE, carboplatin, etoposide, and ifosfamide; IVAC, high‐dose cytarabine, ifosfamide, etoposide, and methotrexate; IVAM, ifosfamide, etoposide, cytarabine, and methotrexate; Pola‐BR, polatuzumab vedotin, bendamustine, and rituximab.

Of the 18 patients who responded to the first‐line salvage chemotherapy, 16 (88.9%) continued to respond to the second‐line salvage chemotherapy, and two (11.1%) had PD. Of the 58 patients with SD or PD after the first‐line salvage chemotherapy, 14 (24.1%) responded to the second‐line salvage chemotherapy, 17 (29.3%) had SD, and 27 (46.6%) had PD. There was no clear relationship between the type of salvage regimen and response rate (Table 3).

**TABLE 3:** Breakdown of responses to second‐salvage chemotherapy.

	Response to second‐line salvage chemotherapy No. (%)			
	CR/PR	SD	PD	*p* value
All patients	30 (39.5)	17 (22.4)	29 (38.2)	
Response to first‐line salvage chemotherapy				< 0.001
CR	6 (100)	0	0	
PR	10 (83.3)	0	2 (16.7)	
SD	4 (19)	12 (57.1)	5 (23.8)	
PD	10 (27)	5 (13.5)	22 (59.5)	
Type of second‐line salvage chemotherapy				0.34
AraC‐based regimen	11 (55)	4 (20)	5 (25)	
Ifosfamide‐based regimen	9 (30)	5 (16.7)	16 (53.3)	
Gemcitabine‐based regimen	3 (23.1)	5 (38.5)	5 (38.5)	
Pola‐BR	5 (55.6)	2 (22.2)	2 (22.2)	
Other	2 (50)	1 (25)	1 (25)	
Patients refractory to first‐line salvage chemotherapy	14 (24.1)	17 (29.3)	27 (46.6)	
Type of second‐line salvage chemotherapy				0.52
AraC‐based regimen	4 (33.3)	4 (33.3)	4 (33.3)	
Ifosfamide‐based regimen	7 (25.9)	5 (18.5)	15 (55.6)	
Gemcitabine‐based regimen	0	5 (50)	5 (50)	
Pola‐BR	2 (33.3)	2 (33.3)	2 (33.3)	
Other	1 (33.3)	1 (33.3)	1 (33.3)	

Abbreviations: CR, complete response; PD, progressive disease; Pola‐BR, polatuzumab vedotin, bendamustine, and rituximab; PR partial response; SD, stable disease.

The response rate to Pola‐BR (polatuzumab vedotin, bendamustine, and rituximab) was 55.6% (5/9) and 33.3% (2/6) in the entire cohort and in patients who failed to respond to the first‐line salvage chemotherapy, respectively (Table 3). These figures were relatively high compared to other regimens (ORR: 37.3% and 23.1%, *p* value: 0.31 and 0.62).

### 3.3 Response to third‐line salvage chemotherapy

After the second‐line salvage chemotherapy, 39 patients received third‐line salvage chemotherapy with curative intent, aiming mainly for CAR T‐cell therapy (three patients received as just a bridge to CAR T‐cell therapy). Eleven patients responded and 28 patients did not respond to second‐line salvage chemotherapy. Among the 11 responders, five changed their chemotherapy regimen owing to renal failure, two were considered inadequate response for proceeding to CAR T‐cell therapy, and four changed their regimen for other reasons. In total, patients received a median of one cycle (range: 1–9) of the third‐line salvage chemotherapy (Table 4). Response was evaluated in 38 patients at data cutoff. Following the third‐line salvage chemotherapy, nine of 38 patients (23.7%) had CR or PR, four (10.5%) had SD, and 25 (65.7%) had PD (Table 5). Out of the 39 patients receiving third‐line salvage chemotherapy, one (1/1 responder) patient proceeded to ASCT, 14 (7/14 responders) patients received CAR‐T and two (1/2 responders) were awaiting CAR T‐cell infusion at the data cutoff.

**TABLE 4:** Details of third‐line salvage chemotherapy.

Characteristic	No. (%)
No. of patients	39
Third‐line salvage chemotherapy	
AraC‐based regimen	
CHASE	5 (12.8)
ESHAP	1 (2.6)
Ifosfamide‐based regimen	
DeVIC	7 (17.9)
Gemcitabine‐based regimen	
GDP	9 (23.1)
GCD	2 (5.1)
Other	
Pola‐BR	15 (38.5)
Median cycle (range)	1 (1–9)
Rituximab in third‐line salvage chemotherapy	37 (94.9)
Response to third‐line salvage chemotherapy	
Complete response or partial response	9/38 (23.7)
Stable disease or progressive disease	29/38 (76.3)
Received potentially curative treatments after third‐line salvage chemotherapy	15 (38.5)

Abbreviations: CHASE, high‐dose cytarabine, cyclophosphamide, etoposide, and dexamethasone; ESHAP, high‐dose cytarabine, cisplatin, etoposide, and methylprednisolone; DeVIC, carboplatin, etoposide, ifosfamide, and dexamethasone; GDP, gemcitabine, cisplatin, and dexamethasone; GCD, gemcitabine, carboplatin, and dexamethasone; Pola‐BR, polatuzumab vedotin, bendamustine, and rituximab.

**TABLE 5:** Breakdown of responses to third‐line salvage chemotherapy.

	Response to third‐line salvage chemotherapy No. (%)			
	CR/PR	SD	PD	*p* value
All patients	9 (23.7)	4 (10.5)	25 (65.7)	
Response to second‐line salvage chemotherapy				0.012
CR	0	1 (50)	1 (50)	
PR	6 (66.7)	0	3 (33.3)	
SD	2 (22.2)	1 (11.1)	6 (66.7)	
PD	1 (5.6)	2 (11.1)	15 (83.3)	
Type of third‐line salvage chemotherapy				0.12
AraC‐based regimen	2 (33.3)	0	4(66.7)	
Ifosfamide‐based regimen	0	0	7 (100)	
Gemcitabine‐based regimen	1 (10)	1 (10)	8 (80)	
Pola‐BR	6 (40)	3 (20)	6 (40)	
Patients refractory to second‐line salvage chemotherapy	3 (11.1)	3 (11.1)	21 (77.8)	
Type of third‐line salvage chemotherapy				0.082
AraC‐based regimen	0	0	2 (100)	
Ifosfamide‐based regimen	0	0	7 (100)	
Gemcitabine‐based regimen	0	0	7 (100)	
Pola‐BR	3 (27.3)	3 (27.3)	5 (45.5)	

Abbreviations: CR, complete response; PD, progressive disease; Pola‐BR, polatuzumab vedotin, bendamustine, and rituximab; PR partial response; SD, stable disease.

Of the 11 patients who responded to the second‐line salvage chemotherapy, six (54.5%) continued to respond to the third‐line salvage chemotherapy, one (9.1%) had SD, and four (36.4%) had PD. Of the 27 patients who had SD or PD after the second‐line salvage chemotherapy, three (11.1%) had CR or PR, three (11.1%) had SD, and 21 (77.8%) had PD. There was also no clear correlation between the type of chemotherapy and response (Table 5).

The response rate to Pola‐BR was 40.0% (6/15) and 27.3% (3/11) in the entire cohort and in patients who failed to respond to the second‐line salvage chemotherapy, respectively (Table 5). These figures were relatively high compared to other regimens (ORR: 13.0% and 0%, *p* value: 0.12 and 0.056).

### 3.4 Survival outcomes after second‐line salvage chemotherapy

**FIGURE 2:**
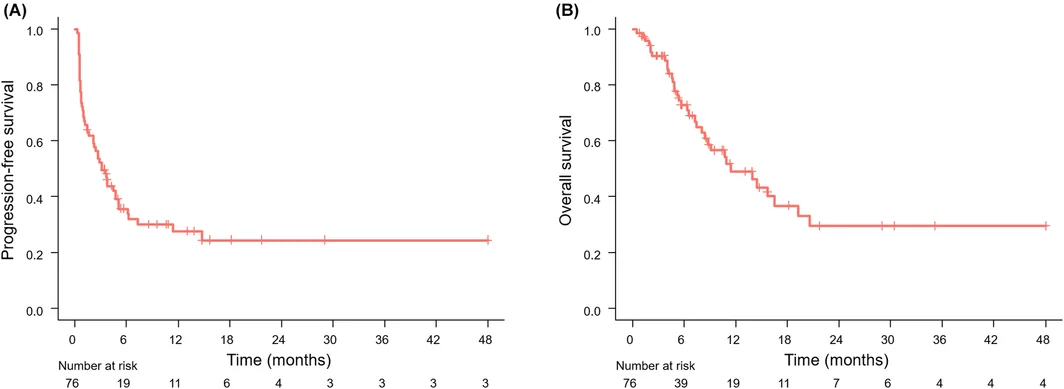
Kaplan–Meier curves of progression‐free survival (A) and overall survival (B) in the entire cohort.

**FIGURE 3:**
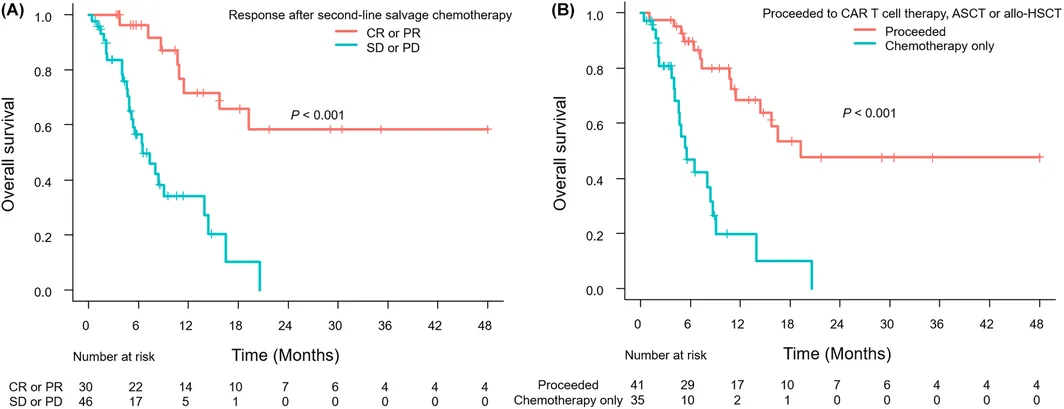
Kaplan–Meier curves of overall survival stratified by the response to second‐line salvage chemotherapy (A) and by whether the patients proceeded to CAR T‐cell therapy, ASCT or allo‐HSCT, or continued chemotherapy only (B). ASCT, autologous hematopoietic stem cell transplantation; CAR, chimeric antigen receptor.

The median follow‐up for survivors was 6.6 months (range: 0.8–90.8 months). Six‐month PFS for the entire cohort after the second‐line salvage chemotherapy was 35.8% (95% confidence interval [CI]: 26.1–49.0), with a median PFS of 3.2 months (95% CI: 2.2–5.1; Figure 2A). Six‐month OS was 72.8% (95% CI: 62.7–84.5), with a median OS of 11.4 months (95% CI: 8.4–20.6; Figure 2B). OS was significantly better in patients who responded to the second‐line salvage chemotherapy than those who did not (median OS: not achieved vs. 6.5 months; *p* < 0.001; Figure 3A). Of all the patients who received second‐line salvage chemotherapy, 41 eventually received a potentially curative treatment (ASCT: 5; CAR T‐cell therapy: 29; allo‐HSCT: 2; ASCT and CAR T‐cell therapy: 5). OS improved significantly in the patients who were able to proceed to the curative treatments than in those who did not (median OS: 19.2 vs. 5.6 months; *p* < 0.001; Figure 3B).

**FIGURE 4:**
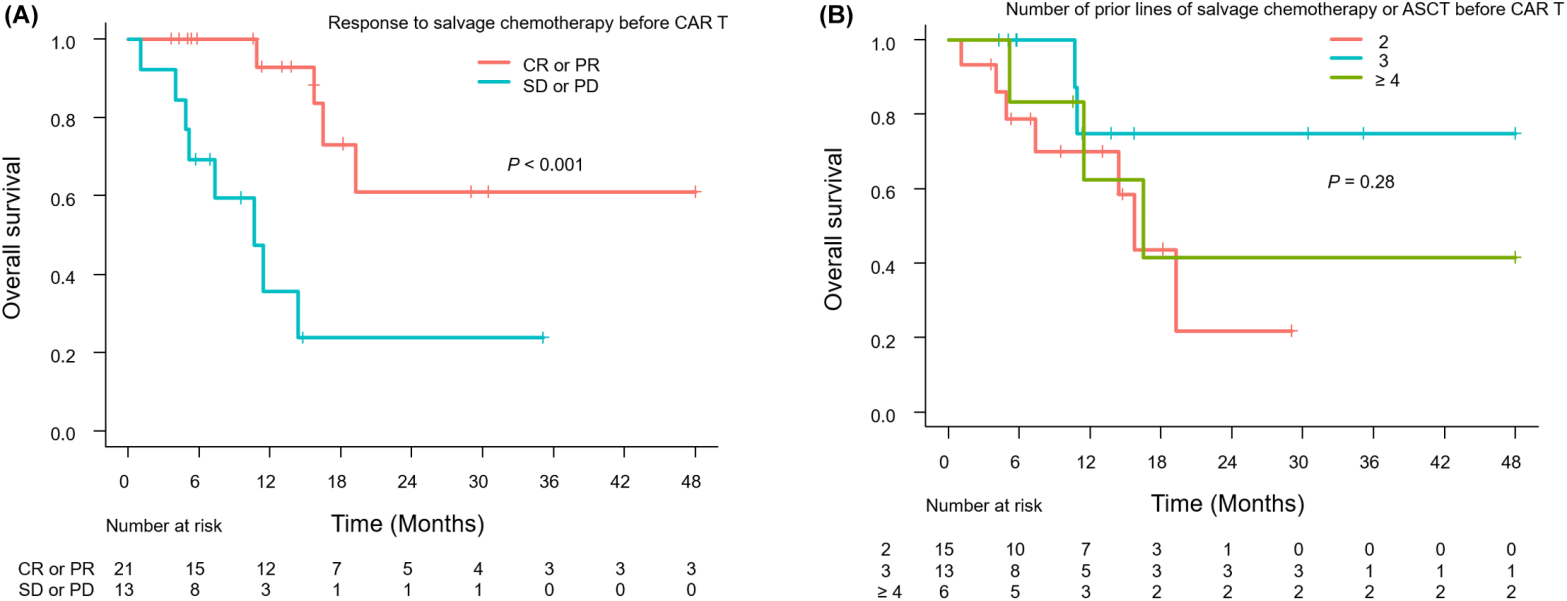
Kaplan–Meier curves of overall survival stratified by the response to chemotherapy immediately prior to CAR T‐cell therapy (A) and by the number of prior lines of salvage chemotherapy or ASCT before CAR T‐cell therapy (B). ASCT, autologous hematopoietic stem cell transplantation; CAR, chimeric antigen receptor.

We analyzed the prognosis of 34 patients who underwent CAR T‐cell therapy. Our findings demonstrated that those who achieved CR or PR following salvage chemotherapy immediately prior to CAR T‐cell therapy had significantly longer OS compared to those with SD or PD (Figure 4A). On the contrary, the number of prior lines of salvage chemotherapy or ASCT before CAR T‐cell infusion did not exhibit any significant association with OS (Figure 4B).

**FIGURE 5:**
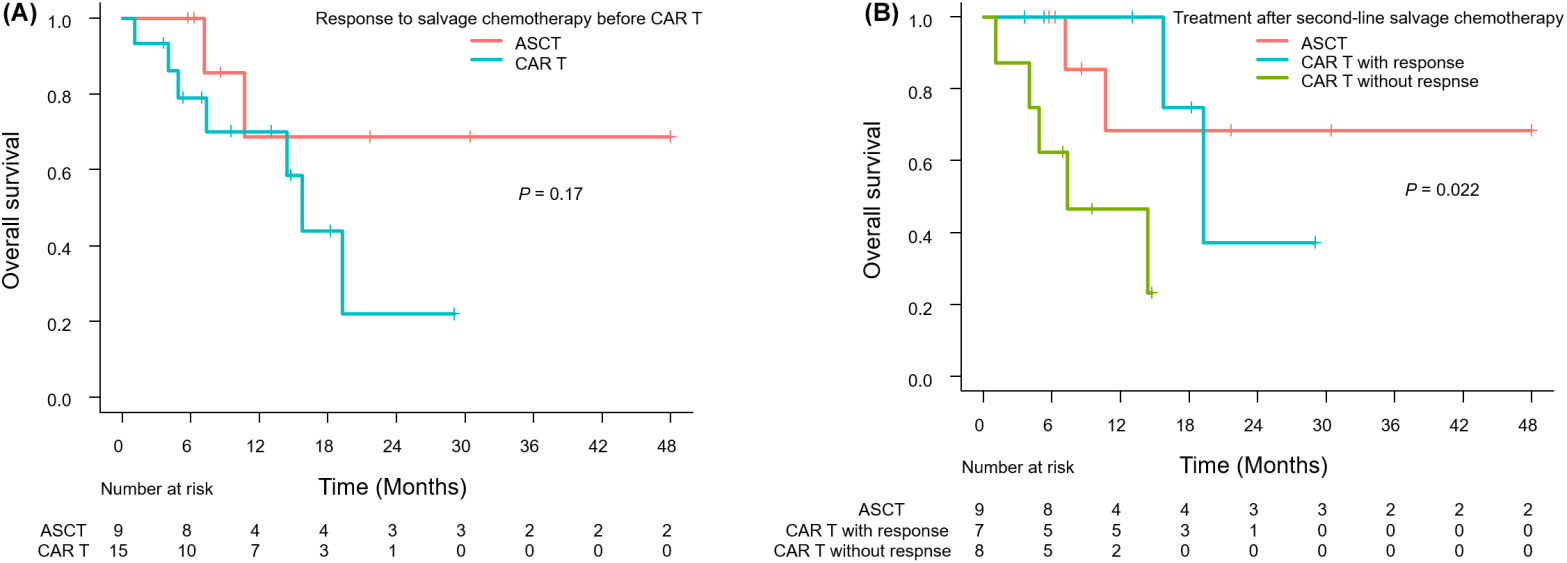
Kaplan–Meier curves of overall survival stratified by whether the patients received CAR T‐cell therapy or ASCT after second‐line salvage chemotherapy (A) and by whether the patients received CAR T‐cell therapy with or without the response to second‐line salvage chemotherapy or ASCT after second‐line salvage chemotherapy (B). ASCT, autologous hematopoietic stem cell transplantation; CAR, chimeric antigen receptor.

While most of the patients who proceeded to ASCT after the second‐line salvage chemotherapy responded to the chemotherapy, only about half the patients who received CAR T‐cell therapy responded to the second‐line salvage chemotherapy (Table S2). However, no significant difference was detected in OS between patients receiving ASCT and those receiving CAR T‐cell therapy (Figure 5A). Furthermore, patients who responded to second‐line salvage chemotherapy prior to receiving CAR T‐cell therapy demonstrated better OS (Figure 5B).

On univariate analysis, significant prognostic factors of PFS and OS were time to relapse, response to first‐line salvage therapy, LDH, extranodal involvement ≥2, and IPI at the time of the second‐line salvage chemotherapy (Table 6). On multivariate analysis, extranodal involvement ≥2 at second‐line salvage therapy was significant prognostic factors of OS (HR 7.10; 95% CI: 1.96–25.7; *p* = 0.003) (Table S3).

**TABLE 6:** Progression‐free survival and overall survival by prognostic factor in patients receiving second‐line salvage chemotherapy.

Factor	Total No. of patients	6‐month PFS		6‐month OS	
		%	*p* value	%	*p* value
All patients	76	35.8		72.8	
Diagnosis					
DLBCL, NOS	56	35.0	0.61	75.5	0.60
Other	20	37.5		65.3	
Cell of origin					
GCB	36	39.3	0.86	67.5	0.78
Non‐GCB	33	38.1		86.8	
Time to relapse					
Relapse, >12 months	14	85.7	0.001	90.0	0.016
Refractory, <12 months	62	24.9		69.3	
Response to first‐line salvage therapy					
CR/PR	18	58.9	0.005	100	0.010
SD/PD	58	28.3		63.1	
LDH > ULN at second‐line salvage therapy					
Yes	42	22	0.008	56.7	0.005
No	22	61.8		94.7	
Stage at second‐line salvage therapy					
I/II	51	34.5	0.79	77.9	0.88
III/IV	25	38.8		61.5	
Extranodal involvement at second‐line salvage therapy					
0–1	64	41.6	<0.001	82.3	<0.001
≥2	12	0		20.2	
IPI score at second‐line salvage therapy					
0–2	60	39.7	0.022	79.3	0.020
≥3	15	16.0		38.6	

Abbreviations: CR, complete response; GCB, germinal center B‐cell; IPI, International Prognostic Index; LDH, lactate dehydrogenase; NA, not available; OS, overall survival; PD, progressive disease; PFS, progression‐free survival; PR, partial response; SD, stable disease; ULN, upper limit of normal.

## 4 DISCUSSION

The present, real‐world study of patients with R/R DLBCL after second‐line salvage chemotherapy demonstrated that the ORR of the second‐line salvage chemotherapy was 39.5%. The patients who responded to the second‐line salvage chemotherapy and those who received ASCT, CAR T‐cell therapy, or allo‐HSCT had good survival outcomes. We demonstrated that chemosensitivity remained a critical factor in predicting survival outcomes following CAR T‐cell therapy, regardless of administration timing. Furthermore, both ASCT and CAR T‐cell therapy are viable therapeutic options after achieving a response to second‐line salvage chemotherapy.

Consolidative high‐dose chemotherapy followed by ASCT has been part of the standard management of R/R DLBCL for the last 25 years.^43^ A response to salvage chemotherapy is essential to proceed to ASCT and is significantly associated with longer survival after transplantation.^44^, ^45^, ^46^ On the other hand, CAR T‐cell therapy may be effective even in cases of chemorefractory DLBCL. However, certain factors, such as the total metabolic tumor volume and active tumor proliferation at the time of lymphodepletion before CAR T‐cell infusion, are associated with progression, early progression, and death in patients with R/R DLBCL.^47^, ^48^ It is thought that disease control with salvage chemotherapy can increase the survival benefit of CAR T‐cell therapy. Therefore, chemosensitivity to salvage regimens is crucial for favorable outcomes in R/R DLBCL patients whether or not they plan to receive ASCT or CAR T‐cell therapy. Our study also demonstrated that the patients achieving CR/PR after the second‐line salvage chemotherapy had longer OS in line with previous reports.^17^, ^18^, ^20^ On the other hand, patients who had disease progression or failed to respond to the second‐line salvage chemotherapy were unlikely to respond to further chemotherapy and had a dismal prognosis.

Randomized studies have produced no clear evidence of the superiority of one salvage regimen over another,^9^, ^11^, ^16^, ^49^ and most clinical trials comparing salvage regimens were performed in the first‐line setting. The literature on the outcomes of second‐line salvage chemotherapy in transplant‐eligible patients is even more limited. The ORR in second‐line salvage chemotherapy ranged from 13.7% to 52% in previous studies (Table S4), the difference probably stemming from the heterogeneity of the patient cohorts and response to first‐line salvage chemotherapy. The ORR in our cohort was 39.5% for all the patients and 24.1% for those with refractory to first‐line salvage chemotherapy, which were comparable to those seen in these reports. On the other hand, the ORR of the first‐line salvage chemotherapy in this cohort was 23.7%, which was lower than the ORR of the second‐line salvage chemotherapy (39.5%). Interestingly, some patients who shifted from one salvage regimen to another (e.g., from AraC‐based regimen to ifosfamide‐based regimen or vice versa) were reported to exhibit notable responses,^18^ which could account for the improved outcome in the second‐line salvage chemotherapy.

The type of salvage regimen was not clearly associated with treatment response in this study. However, the response rate to Pola‐BR seemed to be higher than to other regimens even in patients who were previously resistant to salvage chemotherapy, although the differences were nonsignificant. A previous cohort study reported that Pola‐BR achieved the best ORR at 48.1% in patients who received a median of three prior treatment lines.^50^ This figure was comparable to the findings of this study (Tables 3 and 5). These results suggested that Pola‐BR may be a feasible treatment option for improving the response to prior salvage chemotherapies in patients with SD/PD. Additionally, we investigated the association of the response of Pola‐BR and cell of origin. As previously reported, patients with DLBCL of non‐GCB origin were more likely to respond to Pola‐BR than those of GCB origin in the entire cohort and in second‐line and third‐line setting, albeit lacking statistical significance (Table 7).^40^ However, careful sequencing of this regimen and CAR T‐cell therapy is warranted, given the risk of prolonged lymphopenia caused by bendamustine, which has the potential to impair T‐cell collection for future CAR T‐cell manufacturing.^51^, ^52^, ^53^ In the present study, six patients underwent leukapheresis after receiving Pola‐BR. Bendamustine was stopped for over 8 weeks before leukapheresis to allow CAR T‐cells to be grown.

**TABLE 7:** Breakdown of responses to Pola‐BR according to cell of origin.

	Response to Pola‐BR No. (%)			
	CR/PR	SD	PD	*p* value
All patients received pola‐BR	11 (43.5)	5 (21.7)	8 (34.8)	
Cell of origin				0.26
GCB	4 (33.3)	4 (33.3)	4 (33.3)	
Non‐GCB	7 (63.6)	1 (9.1)	3 (27.3)	
Missing data	0	0	1	
Patients received Pola‐BR as second‐line salvage chemotherapy				
Cell of origin				0.16
GCB	1 (25)	1 (25)	2 (50)	
Non‐GCB	4 (80)	1 (20)	0	
Patients received Pola‐BR as third‐line salvage chemotherapy				
Cell of origin				0.23
GCB	3 (37.5)	3 (37.5)	2 (25)	
Non‐GCB	3 (50)	0	3 (50)	
Missing data	0	0	1	

Abbreviations: CR, complete response; GCB, germinal center B‐cell type; PD, progressive disease; Pola‐BR, polatuzumab vedotin, bendamustine, and rituximab; PR, partial response; SD, stable disease.

In the present study, several clinical factors significantly affected PFS after the second‐line salvage chemotherapy, including the time to relapse, response to the first‐line salvage therapy, LDH, extranodal involvement, and IPI at the second‐line salvage therapy. These prognostic factors have been examined in previous studies in patients who received first‐line salvage chemotherapy.^9^, ^54^, ^55^ To overcome these adverse prognostic factors, new treatment strategies, including novel drugs, are needed.

As illustrated in Figure 4, the response to salvage chemotherapy was a significant prognostic factor in patients receiving CAR T‐cell therapy, although the number of previous chemotherapy regimens exhibited no significant correlation with survival outcome. This result may suggest that maintaining disease control before CAR T‐cell infusion carries greater significance than early infusion of CAR T‐cell. However, we should take into account that the response rate of salvage chemotherapy diminishes as the number of lines increases. Furthermore, we compared the prognosis of patients with relapse subsequent to first‐line salvage therapy and ASCT versus those undergoing ASCT or CAR T‐cell therapy after second‐line salvage regimen. Notably, there were no statistically significant differences observed between the two groups (Figure S1).

Although high‐dose chemotherapy followed by ASCT has been the standard therapy for patients who respond to first‐line salvage chemotherapy, its role after second‐line salvage chemotherapy remains unclear. CAR T‐cell therapy is being increasingly used in patients who are refractory to first‐line salvage chemotherapy. However, as of yet there are few direct analyses comparing CAR T‐cell therapy with ASCT in the post‐second‐line salvage chemotherapy setting. Given that the efficacy of ASCT predominantly depends on chemosensitivity and that the response rate to chemotherapy generally decreases after each line of chemotherapy, not many patients benefit from ASCT after second‐line or subsequent salvage chemotherapy. However, a recent, retrospective, cohort study reported the encouraging finding of a 1‐year OS rate of 74% in patients who underwent ASCT after two or more lines of salvage chemotherapy.^56^ On the other hand, chemosensitivity has less influence on the efficacy of CAR T‐cell therapy, which remains a good option for patients who are refractory to salvage chemotherapy. In our study, the patients who received ASCT and those who received CAR T‐cell therapy after the response to second‐line salvage chemotherapy both demonstrated favorable OS. Therefore, ASCT remains a viable alternative for patients with R/R DLBCL even after second‐line salvage chemotherapy if they are responsive to the chemotherapy. Additionally, CAR T‐cell therapy represents a promising alternative for these patients.

In our study, although more patients who received CAR T‐cell therapy were refractory to the second‐salvage chemotherapy than those who underwent ASCT, the prognosis of patients who received CAR T‐cell therapy was not significantly different from that of those who underwent ASCT. CAR T‐cell therapy may have the potential for superior clinical outcomes compared to ASCT. Actually, three randomized trials compared the efficacy of CAR T‐cell therapy with ASCT as second‐line therapy for DLBCL and reached different conclusions.^57^, ^58^, ^59^ The ZUMA‐7 and TRANSFORM studies demonstrated the superiority of second‐line CAR T‐cell therapy over high‐dose chemotherapy followed by ASCT in patients refractory to or relapsing within 12 months of the first‐line anthracycline‐based chemoimmunotherapy.^57^, ^58^ Based on these two trials, axicabtagene ciloleucel and lisocabtagene maraleucel were approved by FDA as second‐line therapy. Given this background, the significance of salvage chemotherapy, especially second or later salvage line of chemotherapy, may diminish. However, patients relapsing after 12 months will receive salvage chemotherapy aiming for ASCT. Furthermore, there is still lack of immediate access to CAR T‐cell therapy in some areas and institutions, and patients have to receive one or more lines of salvage chemotherapy in order to control lymphoma even if they are indicated for CAR T‐cell therapy as second‐line therapy.

The present study had some limitations. This was a retrospective study enrolling a small cohort with a relatively short follow‐up period. In particular, a treatment bias was induced by the physicians determining treatment decisions at their own discretion. However, it included a group of patients who received salvage immunochemotherapy in the same clinical setting. We anticipate that there will still be patients who will receive second‐line salvage chemotherapy for some reasons described above, even if CAR T‐cell therapy is viewed as the superior treatment option as second‐line therapy. We believe that this study can offer some useful insights into the management of R/R DLBCL patients.

## 5 CONCLUSIONS

In conclusion, our results suggested that the response to second‐line salvage chemotherapy was important to proceed to curative treatments and to improve clinical outcomes in patients with R/R DLBCL. The efficacy of CAR T‐cell therapy was found to be influenced by the responsiveness to salvage regimens, regardless of when CAR T‐cell therapy was administered. Moreover, our study suggested that the favorable OS was observed in patients who underwent ASCT or CAR T‐cell therapy following the response to the second‐line salvage chemotherapy, suggesting that ASCT and CAR T‐cell therapy were viable treatment options. However, the ORR was still insufficient, especially in patients who were refractory to prior salvage regimens. Improvement of the efficacy of salvage chemotherapy and refinement of treatment strategies for R/R DLBCL are urgent issues that await addressing.

## AUTHOR CONTRIBUTIONS

**Yu Yagi:** Conceptualization (lead); resources (lead); writing – original draft (lead). **Yusuke Kanemasa:** Resources (supporting); writing – review and editing (lead). **Yuki Sasaki:** Data curation (lead). **Mina Sei:** Resources (supporting). **Takuma Matsuo:** Resources (supporting). **Kento Ishimine:** Resources (supporting). **Yudai Hayashi:** Resources (supporting). **Mano Mino:** Resources (supporting). **An Ohigashi:** Resources (supporting). **Yuka Morita:** Resources (supporting). **Taichi Tamura:** Resources (supporting). **Shohei Nakamura:** Resources (supporting). **Toshihiro Okuya:** Resources (supporting). **Takuya Shimizuguchi:** Resources (supporting). **Naoki Shingai:** Resources (supporting). **Takashi Toya:** Resources (supporting). **Hiroaki Shimizu:** Resources (supporting). **Yuho Najima:** Resources (supporting). **Takeshi Kobayashi:** Resources (supporting). **Kyoko Haraguchi:** Resources (supporting). **Noriko Doki:** Resources (supporting). **Yoshiki Okuyama:** Resources (supporting). **Tatsu Shimoyama:** Conceptualization (lead); resources (lead); supervision (lead).

## CONFLICT OF INTEREST STATEMENT

Noriko Doki—Lecture Fees: Janssen, Novartis.

## ETHICS STATEMENT

The present, retrospective study was performed in accordance with the Declaration of Helsinki and was approved by the Ethics Committee of Tokyo Metropolitan Cancer and Infectious Diseases Center, Komagome Hospital.

## PATIENT CONSENT STATEMENT

Written informed consent was waived because the present study used clinical data retrospectively obtained from the hospital medical records.

## Supporting information


Figure S1.

Table S1.

Table S2.

Table S3.

Table S4.

## Data Availability

The data generated during and/or analyzed in the current study are available from the corresponding author on reasonable request.
