# Supplementary figures and images for: Clinical outcomes in transplant‐eligible patients with relapsed or refractory diffuse large B‐cell lymphoma after second‐line salvage chemotherapy: A retrospective study
Source: Cancer Med. 2023 Aug 28;12(17):17808–21. doi: 10.1002/cam4.6412 (PMC10523963; doi:10.1002/cam4.6412)

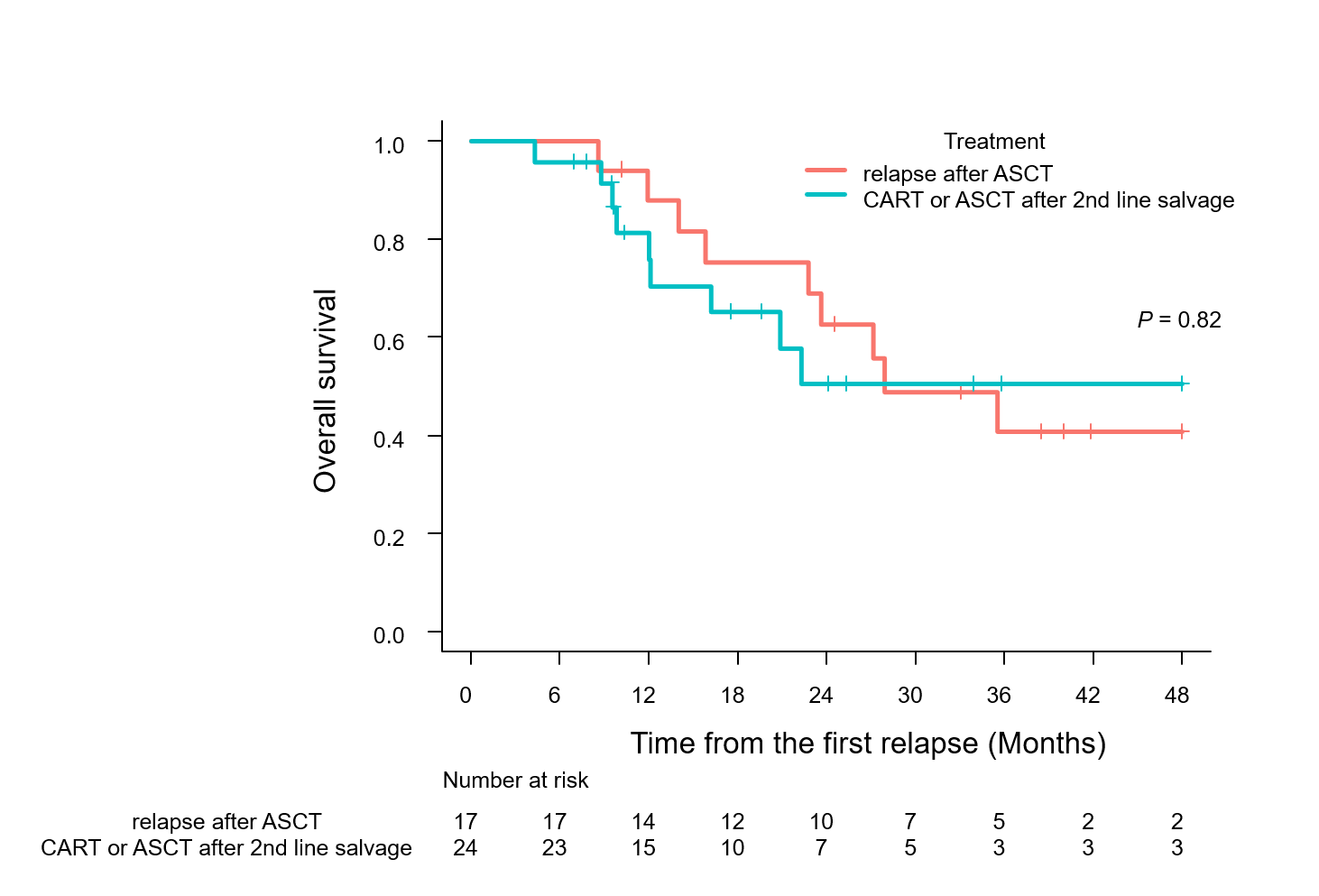

Supplement: Supplementary file 1 — Figure S1. [file CAM4-12-17808-s001.tif]
