# Supplementary material for: Clinical outcomes in transplant‐eligible patients with relapsed or refractory diffuse large B‐cell lymphoma after second‐line salvage chemotherapy: A retrospective study
Source: Cancer Med. 2023 Aug 28;12(17):17808–21. doi: 10.1002/cam4.6412 (PMC10523963; doi:10.1002/cam4.6412)
Supplement: Supplementary file 2 — Table S1. [file CAM4-12-17808-s003.docx]

Supplementary Table 1. Salvage regimens used in this study

| Regimen | Drugs | Reference |
| --- | --- | --- |
| ESHAP | Cisplatin 25 mg/m^2^ and etoposide 40 mg/m^2^ on days 1–4; methyl prednisolone 500 mg/d on days 1–5; cytarabine 2 g/m^2^ on day 5 | 26 |
| DHAP | Cisplatin 100 mg/m^2^ on day 1; cytarabine 2 g/m^2^ twice on day 2; dexamethasone 40 mg on days 1–4 | 27 |
| CHASE | Cyclophosphamide 1200 mg/m^2^ on day 1; etoposide 100 mg/m^2^ and dexamethasone 40mg on days 1–3; cytarabine 2 g/m^2^ on days 2–3 | 28 |
| ACES | Carboplatin 100 mg/m^2^ and etoposide 80 mg/m^2^ on days 1–4; solumedrol 500 mg on days 1–5; cytarabine 2 g/m^2^ on day 5 | 29 |
| HyperCVAD | Cyclophosphaide 300mg/m2 on day 1–3; doxorubicin 16.6 mg/m^2^ on day 4–6; vincristine 1.4 mg/m^2^ on day 4; dexamethasone 40mg days 1–4; cycle 1, 3, and 5. MTX 1000mg/m^2^ on day 1; cytarabine 3 g/m^2^ on day2 and 3; cycle 2, 4, 6. | 30 |
| ICE | Etoposide 100 mg/m^2^ on days 1–3; ifosfamide 5 g/m^2^ and carboplatin area under the curve 5, capped at 800 mg on day 2 | 31 |
| DeVIC | Carboplatin 300 mg/m^2^ on day 1; ifosfamide 1.5 g/m^2^, etoposide 100 mg/m^2^, and dexamethasone 40 mg on days 1–3 | 32 |
| IVAC | Ifosfamide 1500 mg/m^2^ and etoposide 60 mg/m^2^ on days 1–5; cytarabine 2 g/m^2^ twice on days 1–2; methotrexate 12 mg i.t. on day 5 | 33 |
| IVAM | Etoposide 150 mg/m^2^ and cytarabine 100 mg/m^2^ on days 1–3; ifosfamide 1500 mg/m^2^ on days 1–5; methotrexate 3 g/m^2^ on day 5 | 34 |
| GDP | Cisplatin 75mg/m^2^ on day 1; gemcitabine 1000 mg/m^2^ on days 1 and 8; dexamethasone 40mg days 1–4 | 35 |
| GCD | Carboplatin AUC 5 on day 1; gemcitabine 1000mg/m^2^ on days 1 and 8; dexamethasone 40mg on days 1–4 | 36 |
| DA–EPOCH | Etoposide 50 mg/m^2^, doxorubicin 10 mg/m^2^, and vincristine 0.4 mg/m^2^ on days 1–4; prednisone 60 mg/m^2^ on days 1–5; cyclophosphamide 750 mg/m^2^ on day 5 | 37 |
| EPOCH | Etoposide 65 mg/m^2^, doxorubicin 15 mg/m^2^ and vincristine 0.5 mg/m^2^ on days 1–4; prednisone 60 mg/m^2^ on days 1–5; cyclophosphamide 750 mg/m^2^ on day 5 | 38 |
| BR | Rituximab 375 mg/m^2^ on day 1; bendamustine 120 mg/m^2^ on days 2–3 | 39 |
| Pola–BR | Polatuzumab vedotin 1.8 mg/kg on day 2 of cycle 1 and day 1 of subsequent cycles; bendamustine 90 mg/m^2^ on days 2–3 of cycle 2, then days 1 and 2 of subsequent cycles; rituximab 375 mg/m^2^ on day 1 of each cycle | 40 |
