# Supplementary material for: Clinical outcomes in transplant‐eligible patients with relapsed or refractory diffuse large B‐cell lymphoma after second‐line salvage chemotherapy: A retrospective study
Source: Cancer Med. 2023 Aug 28;12(17):17808–21. doi: 10.1002/cam4.6412 (PMC10523963; doi:10.1002/cam4.6412)
Supplement: Supplementary file 3 — Table S2. [file CAM4-12-17808-s002.docx]

Supplementary Table 2. Characteristics of patients receiving ASCT or CAR T-cell therapy after second-line salvage chemotherapy

| Characteristic | ASCT no. (%) | CAR T no. (%) | *P* value |
| --- | --- | --- | --- |
| No. of patients | 9 | 15 |  |
| Refractory relapse, <12 | 7 (77.8) | 12 (80) | 1 |
| CR/PR after second-line salvage therapy | 8 (88.9) | 7 (46.7) | 0.10 |
| CR/PR after first-line salvage therapy | 5 (55.6) | 3 (20) | 0.18 |
| LDH > ULN at second-line salvage therapy | 1/5 (20) | 8/14 (57.1) | 0.37 |
| ≥ 2 Extranodal involvement at second-line salvage therapy | 0 | 3 (20) | 0.43 |
| ≥ 3 IPI at second-line salvage therapy | 0 | 2 (13.3) | 0.76 |

*ASCT* autologous stem cell transplantation, *CAR* chimeric antigen receptor, *CR* complete response, *PR* partial response, *LDH* lactate dehydrogenase, *ULN* upper limit of normal, *IPI* International Prognostic Index
