# Supplementary material for: Clinical outcomes in transplant‐eligible patients with relapsed or refractory diffuse large B‐cell lymphoma after second‐line salvage chemotherapy: A retrospective study
Source: Cancer Med. 2023 Aug 28;12(17):17808–21. doi: 10.1002/cam4.6412 (PMC10523963; doi:10.1002/cam4.6412)
Supplement: Supplementary file 4 — Table S3. [file CAM4-12-17808-s004.docx]

Supplementary Table 3. Multivariate analysis of progression-free survival and overall survival after second salvage chemotherapy

| Factor | PFS | | OS | |
| --- | --- | --- | --- | --- |
|  | Hazard ratio (95% CI) | *P* value | Hazard ratio (95% CI) | *P* value |
| Refractory, < 12 months | 2.98 (0.83–10.7) | 0.093 | 2.74 (0.52–14.4) | 0.23 |
| SD/PD to first-line salvage therapy | 1.89 (0.72–4.95) | 0.20 | 1.67 (0.53–5.3) | 0.38 |
| LDH > ULN at second-line salvage therapy | 1.58 (0.73–3.41) | 0.25 | 2.54 (0.95–6.81) | 0.064 |
| Extranodal sites ≥ 2 at second-line salvage therapy | 2.37 (0.77–7.28) | 0.13 | 7.10 (1.96–25.7) | 0.003 |
| IPI score ≥ 3 at second-line salvage therapy | 1.22 (0.41–3.58) | 0.73 | 2.22 (0.69–7.2) | 0.18 |

*PFS* progression–free survival, *OS* overall survival, *CI* confidence interval, *SD* stable disease, *PD* progressive disease, *LDH* lactate dehydrogenase, *ULN* upper limit of normal, *IPI* International Prognostic Index,
