# Supplementary material for: Clinical outcomes in transplant‐eligible patients with relapsed or refractory diffuse large B‐cell lymphoma after second‐line salvage chemotherapy: A retrospective study
Source: Cancer Med. 2023 Aug 28;12(17):17808–21. doi: 10.1002/cam4.6412 (PMC10523963; doi:10.1002/cam4.6412)
Supplement: Supplementary file 5 — Table S4. [file CAM4-12-17808-s005.docx]

Supplementary Table 4. Comparison of the results of the current study with those of previous studies of second-line salvage chemotherapy

|  | Current study | Rassy, E., et al | Van Den Neste, E., et al | Seshadri, T., et al | Simpson, L., et al | Jhatial, M.A., et al | Elstrom, R.L., et al | Ardeshna K.M, et al |
| --- | --- | --- | --- | --- | --- | --- | --- | --- |
| Reference no. |  | 17 | 18 | 19 | 20 | 21 | 22 | 23 |
| Study design | Retrospective, single-center | Retrospective, single-center | A subset of the phase Ⅲ study, multi-center | Retrospective, multi-center | Retrospective, single-center | Retrospective, single-center | Retrospective, two-center | Retrospective, two-center |
| Patients number | 53 | 46 | 203 | 73 | 21 | 52 | 21 | 57 |
| Diagnosis |  |  |  |  |  |  |  |  |
| DLBCL NOS | 38 (71.7) | 46 (100) | 203 (100) | 54 (74) | 21 (100) | 52 (100) | 21 (100) | 0 |
| Transformed | 7 (13.1) | 0 | 0 |  | 0 | 0 | 0 | 3 (5.3) |
| Other | HGBL; 3 (5.7)  PMBCL; 4 (7.6)  IVLBCL; 1 (1.9) | 0 | 0 | PMBCL; 19 (26) | 0 | 0 | 0 | HL; 17 (29.8)  NHL; 26 (45.6)  Indolent-NHL; 11 (19.3) |
| Cell of origin |  |  |  |  |  |  |  |  |
| GCB | 25 (47.2) | 19 (41.3) | 48 (47.1) | NA | NA | NA | NA | NA |
| Non-GCB | 21 (39.6) | 7 (15.2) | 54 (52.9) | NA | NA | NA | NA | NA |
| Unknown | 7 (13.2) | 20 (43.5) | 101 | NA | NA | NA | NA | NA |
| Refractory or relapsed <12 months to first-line therapy | 42 (79.3) | 32 (69.6) | 117 (57.6) | Refractory; 43(58.9) | Refractory; 12 (57.1) | 48 (92.3) | Refractory; 17 (81) | Refractory; 34 (59.6) |
| First-line salvage chemotherapy | ESHAP-like; 23 (43.4)  CHASE; 16 (30.2)  GDP; 7 (13.2)  ICE-like; 4 (7.5)  Other; 3 (5.7) | R-DHAP; 39 (84.8)  R-ICE; 7 (15.2) | DHAP; 94 (46.3)  R-ICE; 109 (53.7) | DHAP; 39(53)  ESHAP; 20 (27)  GDP; 14(20) | DHAP | ICE; 37 (71)  DHAP; 12 (23)  Other; 3 (6) | NA | ESHAP; 51(89.4)  MiniBEAM; 4  Other; 2 (10.5) |
| Second-line salvage chemotherapy | ESHAP-like; 6 (11.3)  ICE-like; 17 (32.1)  Gemcitabine-containing; 9 (17)  CHASE; 9 (17)  Other; 12 (22.6) | R-DHAP; 7 (15.2)  R-ICE; 39 (84.8) | ICE-like; 31 (18.5)  DHAP-like; 30 (18)  Gemcitabine-containing; 23 (13.8)  Other; 82 (40.4) | GDP; 4 (5)  ESHAP; 7(10)  ICE/DICE; 15(21)  MiniBEAM; 39(53)  IVAM; 6(8)  VACOP-B; 2(3) | ICE±R | Gemcitabine- containing | PEP-C; 6 (22)  Methotrexate/cytarabine; 5(19)  (R) DICE; 2(8)  (R) ESHAP; 2 (8) | DHAP; 2 (3.5)  ESHAP; 4 (7.0)  miniBEAM; 34 (59.6)  IVE ;14 (24.6)  Other; 3(5.3) |
| ORR | 21 (41.2) | 15 (32.6) | 79/166 (47.6) | 10(13.7) | 11 (52) | 14 (26.9) | 5 (23.8) | 18 (32) |
| ORR in patients refractory to first-line salvage chemotherapy | 11/39 (28.2) | 7/33 (21.2) | 43/112 (38.4) | 10(13.7) | 11 (52) | NA | NA | 6/39 (15.4) |
| Advancement to transplantation or CAR-T cell therapy | ASCT; 7 (13.2)  Allo-HSCT; 2 (3.8)  CAR-T; 11 (20.8) | ASCT; 12 (26.1) | ASCT; 56 (27.6)  Allo-HSCT; 8 (4.0) | ASCT; 8(11.0) | ASCT; 9 (43) | ASCT; 2 (3.9) | NA | 25 (43.8) |
| Median follow-up (months, range) | 5.4 (0.4–90.1) | 25.2 | NA | 1.4 (0.6-7.2) years | NA | NA | NA | 46 |
| Median PFS (months, range) | 2.2 (0.90–3.7) | 2.1 (1.8–4.1) | NA | NA | NA | 4.0 | NA | 4 (3-14) |
| Median OS (months, range) | 8.0 (5.6–NE) | 11.4 (9.5–42.7) | 4.4 | NA | NA | 13.0 | NA | 15 (9-26) |

*DLBCL* diffuse large B-cell lymphoma, *NOS* not otherwise specified, *HGBCL* high-grade B-cell lymphoma, *PMBCL* primary mediastinal large B-cell lymphoma, *IVLBCL* intravascular large B-cell lymphoma, *TFL* transformed follicular lymphoma, *HL* Hodgkin’s lymphoma, *NHL* non-Hodgkin’s lymphoma, *GCB* germinal center B-cell type, *NA* not available, *ESHAP* high-dose cytarabine, cisplatin, etoposide, and methylprednisolone, *CHASE* high-dose cytarabine, cyclophosphamide, etoposide, and dexamethasone, *GDP* gemcitabine, cisplatin, and dexamethasone, *ICE* carboplatin, etoposide, and ifosfamide, *DHAP* high-dose cytarabine, cisplatin, and dexamethasone, *BEAM* carmustine, etoposide, cytarabine, melphalan, *DICE* carboplatin, etoposide, ifosfamide, and dexamethasone, IVAM ifosfamide, etoposide, cytarabine, and methotrexate, *VCOP-B* etoposide, doxorubicin, cyclophosphamide, vincristine, prednisone, bleomycin, PEP-C procarbazine, etoposide, prednisone, cyclophosphamide, *IVE* ifomide, etoposide, and epirubicin, *ORR* overall response rate, *CAR-T* chimeric antigen receptor, *ASCT* autologous stem cell transplant, Allo-HCT allogenic hematopoietic stem cell transplantation, PFS progression-free survival, OS overall survival
